# Complete PHB mobilization in *Escherichia coli *enhances the stress tolerance: a potential biotechnological application

**DOI:** 10.1186/1475-2859-8-47

**Published:** 2009-08-31

**Authors:** Qian Wang, Hongmin Yu, Yongzhen Xia, Zhen Kang, Qingsheng Qi

**Affiliations:** 1 State Key Laboratory of Microbial Technology, National Glycoengineering Research Center, School of Life Science, Shandong University, Jinan, 250100 PR China

## Abstract

**Background:**

Poly-*β*-hydroxybutyrate (PHB) mobilization in bacteria has been proposed as a mechanism that can benefit their host for survival under stress conditions. Here we reported for the first time that a stress-induced system enabled *E. coli*, a non-PHB producer, to mobilize PHB *in vivo *by mimicking natural PHB accumulation bacteria.

**Results:**

The successful expression of PHB biosynthesis and PHB depolymerase genes in *E. coli *was confirmed by PHB production and 3-hydroxybutyrate secretion. Starvation experiment demonstrated that the complete PHB mobilization system in *E. coli *served as an intracellular energy and carbon storage system, which increased the survival rate of the host when carbon resources were limited. Stress tolerance experiment indicated that *E. coli *strains with PHB production and mobilization system exhibited an enhanced stress resistance capability.

**Conclusion:**

This engineered *E. coli *with PHB mobilization has a potential biotechnological application as immobilized cell factories for biocatalysis and biotransformation.

## Background

A wide variety of microorganisms are able to accumulate polyhydroxyalkanoates (PHAs) as intracellular carbon/energy storage compounds or reducing power for coping with changing, often oligotrophic environments [1,2]. Various PHAs, as well as the best-known poly 3-*β*-hydroxybutyrate (PHB), were found to be accumulated and degraded as required under environmental conditions by most natural PHAs producing bacteria [3]. When the environment is sufficient with carbon source or the C/N ratio is quite high (>20), the PHAs accumulation is much faster than degradation [4,5]. While facing different stresses, such as low nutrient availability and detrimental physical, chemical, or biological factors, these bacteria begin to mobilize PHAs to conquer those unfavorable environments. The biosynthesis and degradation of PHAs is a cyclic mechanism that has already been found in many bacteria, such as *Ralstonia eutropha*, *Azotobacter beijerinckii *and *Hydrogenomonas eutropha *[6-8]. The *in vivo *PHB biosynthesis pathway is conducted by the successive action of *β*-ketoacyl-CoA thiolase (*phb*A), acetoacetyl-CoA reductase (*phb*B) and PHB polymerase (*phb*C). However, PHB degradation, which has been investigated for years, was divided into intracellular mobilization and extracellular degradation. Intracellular PHB mobilization is initialized by the hydrolization action of intracellular PHB depolymerase [9,10]. The depolymerized product, (*R*)-3-hydroxybutyric acid (3HB) [11], is then metabolized *in vivo *as carbon and energy source by cells or excreted into the environment. To metabolize (*R*)-3-hydroxybutyric acid, cells have to convert it to acetoacetate by (*R*)-3-hydroxybutyric acid dehydrogenase [12] or activate it to a CoA derivative by enzymes such as acyl-CoA synthetase or thioesterase [13-15]. Acetoacetate can be converted to two molecules acetyl-CoA under the function of *β*-ketothiolase by primarily activated to acetoacetyl-CoA [16], then acetyl-CoA is further metabolized via the tricarboxylic acid (TCA) cycle or the glyoxylate cycle; while (*R*)-3-hydroxybutyl-CoA can be immediately epimerized to the (*S*)-isomer in order to be catabolized by *β*-oxidation pathway for energy release.

*Escerichia coli*, which possesses neither PHB synthase nor depolymerase genes, was thought to be one of the best PHA production candidates. By metabolic engineering, recombinant *E. coli *was confirmed to accumulate PHB up to 90% of dry cell weight [6,17,18]. When co-expressed with *phaZ*1 gene from *R. eutropha*, recombinant *E. coli *was able to depolymerize PHB and secrete 3HB into the medium [9,19]. However, can *E. coli *realize the complete PHB mobilization *in vivo*? Can *E. coli *obtain any benefit on survival in stress conditions from the PHB mobilization as described for several wild type species e.g. *Azospirillum brasilense *and *Sinorhizobium meliloti*? To answer these questions, we constructed a stress induced PHB mobilization system in *E. coli *by mimicking the natural PHB producer. The stress-induced system was developed by introducing 5'-untranslated region of *rpoS *[20]. This stress-induced region (SIR) fragment promotes the transcription of *rpoS *gene, which is induced under stationary phase or under stress conditions [21]. The engineered *E. coli *was then investigated for its stress resistance capability.

## Results

### The complete PHB mobilization in engineered *E. coli*

**Figure 1:**
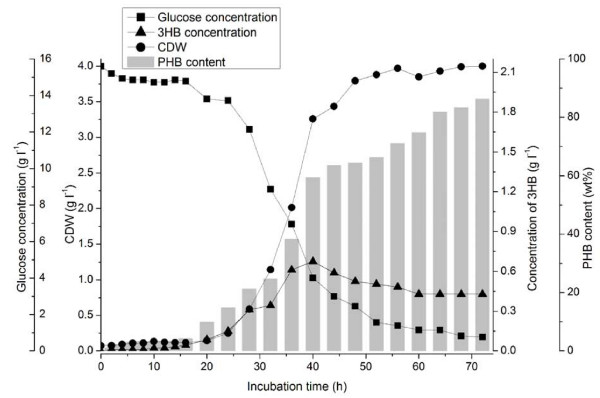
**Cultivation of PHB mobilization strain *E. coli *DH5α (pQWQ2/pSCP-CAB)**. (Filled circle) cell dry weight (CDW); (filled square) glucose consumption; (gray bars) PHB content; (filled triangle) extracellular 3HB concentration.

To mimic the PHB metabolic cycle in *E. coli*, we first constructed a PHB accumulation strain by introducing a stress induced PHB production system in *E. coli *DH5α. The engineered *E. coli *DH5α (pSCP-CAB), which contained the PHB operon and a stress induced promoter, was confirmed to produce PHB in glucose medium at the late exponential phase without adding any inducer (Additional file 1, Fig. S1). Fluorescence microscopy analysis showed that during PHB accumulation, the engineered *E. coli *DH5α was deformed and was longer than normal *E. coli *cells. To realize PHB mobilization in *E. coli *DH5α (pSCP-CAB), the *phaZ*1 gene from *Ralstonia eutropha *was introduced using again the stress induced system. The expression of *phaZ*1 in *E. coli *was confirmed by SDS page (data not shown) and by 3HB releasing analysis of the engineered strain *E. coli *DH5α (pQWQ2/pSCP-CAB) (Fig. 1).

**Figure 2:**
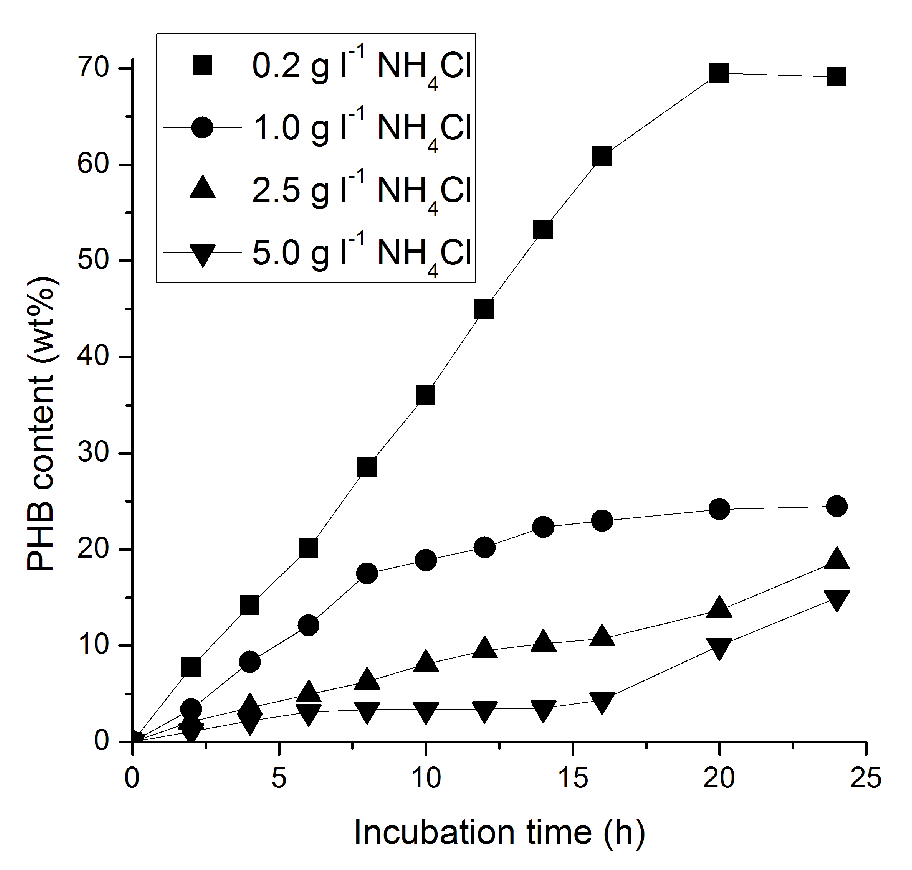
**Induction of PHB accumulation in engineered *E. coli *DH5α (pQWQ2/pSCP-CAB) by nitrogen limitation**.

In natural PHB accumulation bacteria, PHB is accumulated and mobilized inside the cells when bacteria are in imbalance of carbon and nitrogen nourishment [1]. Nitrogen availability is a limiting factor for bacterial growth, especially in some nitrogen-poor environments. These conditions of suboptimal growth are conducive to the production of PHAs [22]. Thus, nitrogen limitation is the key factor for PHB biosynthesis induction in natural PHB production bacteria. To investigate if the nitrogen limitation also induce PHB production in engineered *E. coli*, various amount of nitrogen (NH_4_Cl, 0.2 g/L, 1 g/L, 2.5 g/L, 5 g/L) was tested in M9 medium supplied with glucose as carbon source. Experiment results showed that low nitrogen concentration led to the fast and high PHB accumulation in our engineered *E. coli *(Fig. 2). The initial PHB accumulation (within 20 hours) was varied from 69% to 15% of cell dry weight with gradually increased nitrogen concentrations. The more NH_4_Cl supplied in the medium, the less PHB accumulated in the cells. This phenomenon indicated that nitrogen source limitation served as a stress and induced the transcription of the stress-induced promoter.

(*R*)-3-hydroxybutyric acid (3HB) secretion was detected in the culture supernatant of *E. coli *DH5α (pQWQ2/pSCP-CAB), indicating the realization of PHB mobilization in the engineered *E. coli *(Fig. 1). The 3HB appeared in the medium after 20 hours cultivation along with the PHB formation without additional induction; then the 3HB concentration increased to 0.72 g/L at 40 h. However, the 3HB secretion rate is lower than PHB accumulation rate since the intracellular PHB was accumulated. After that, the 3HB concentration decreased slowly to 0.45 g/L. The slow decrease of 3HB concentration in the medium implied the re-utilization of 3HB by the cells at certain conditions. In recombinant *E. coli *without a *phaZ*1 gene, only little 3HB was detected (0.003 g/L) in culture medium.

To confirm the capability of 3HB utilization by *E. coli*, an *in vitro *experiment of 3-hydroxybutyral-CoA synthetase activity measurement was performed. When the *E. coli *crude enzyme extract was incubated with CoA, ATP, MgCl_2_, DTT and 3HB at 37°C, a depletion of 3HB was observed using HPLC analysis (Table 1). When any of the reacting compounds, such as CoA, ATP, MgCl_2 _or DTT, was omitted from the reaction mixture or when the *E. coli *crude enzyme extract was treated at 95°C for 5 min prior to the assay, there was almost no depletion of 3HB was detected. This demonstrated that an enzymatic activity of acyl-CoA synthetase existed and 3HB can be converted to 3HB-CoA *in vivo*. The consumption of 3HB as carbon and energy source indicated that the complete PHB mobilization in engineered *E. coli *was realized.

**Table 1:** *In vitro *3-hydroxybutyral-CoA synthetase activity assay

Conditions	Concentration of 3HB (mM) at:		
	
	0 h	6 h	12 h
*E.coli *enzyme extract, 3HB, CoA	3.99 ± 0.06	3.86 ± 0.10	3.86 ± 0.06
*E.coli *enzyme extract, 3HB, ATP	3.33 ± 0.02	3.41 ± 0.04	3.29 ± 0.05
*E.coli *enzyme extract, 3HB, ATP, CoA	4.39 ± 0.03	2.02 ± 0.02	0.15 ± 0.10

### PHB mobilization enabled the host for long term starvation

**Figure 3:**
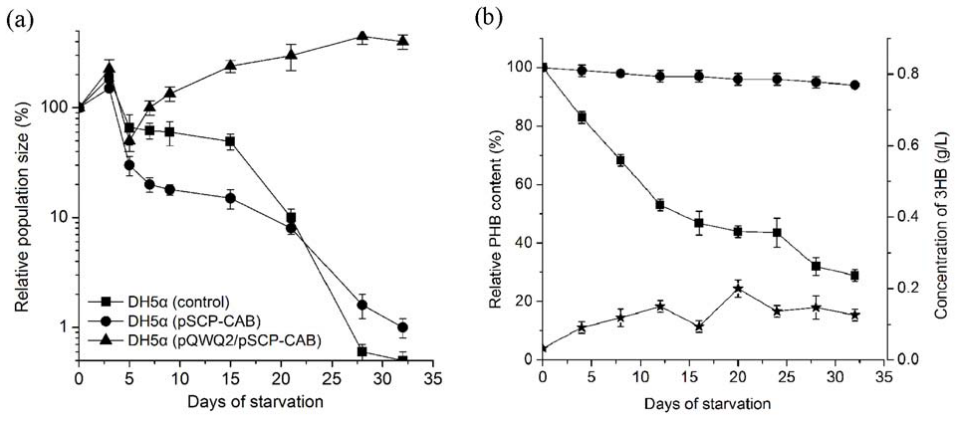
**Effect of PHB mobilization on the survival of starved *E. coli *strains**. (**a**) Relative survival rate. (Filled square) *E. coli *DH5α; (filled circle) *E. coli *DH5α (pSCP-CAB); (filled triangle) *E. coli *DH5α (pQWQ2/pSCP-CAB). (**b**) PHB consumption and 3HB secretion during starvation. (Filled circle) relative PHB content of *E. coli *DH5α (pSCP-CAB); (filled triangle) relative PHB content of *E. coli *DH5α (pQWQ2/pSCP-CAB); (filled pentacle) 3HB secretion of *E. coli *DH5α (pQWQ2/pSCP-CAB).

In order to confirm the complete PHB mobilization in engineered *E. coli*, all the strains were examined for long term starvation in M9 medium without carbon source (32 days). Fig 3A showed that the cell number of all strains began to reduce gradually from the third day of the starvation except the PHB mobilization strain *E. coli *DH5α (pQWQ2/pSCP-CAB), which exhibited an obvious multiplication during starvation. At the end of the starvation, the cell population of *E. coli *DH5α (pQWQ2/pSCP-CAB) increased by three-fold while the other strains without a PHB mobilization system had a under 1% of survival (Fig. 3A). Only about 4% of the cells survived when *E. coli *DH5α (pQWQ2/pSCP-CAB) was incubated in potassium phosphate buffer without nitrogen source or carbon source (Additional file 1, Fig. S2). This result proved the necessity of nitrogen for the survival and multiplication during the starvation. After being starved for 32 days, *E. coli *DH5α (pQWQ2/pSCP-CAB) consumed 72% of its intracellular PHB, indicating the re-utilization of PHB as carbon and energy source (Fig. 3B). During the starvation experiment, we found that the free 3HB of *E. coli *DH5α (pQWQ2/pSCP-CAB) in the medium was maintained at a low level of about 0.1-0.2 g/L. Calculation of the PHB degradation revealed that totally about 0.8 g/L 3HB was produced by depolymerization of PHB during the starvation. This indicated that *E. coli *DH5α (pQWQ2/pSCP-CAB) consumed 0.6 g/L 3HB for carbon and energy. This data further confirmed the existing of PHB cycle in *E. coli *DH5α (pQWQ2/pSCP-CAB).

### Resistance of engineered *E. coli *to different environmental stress

**Figure 4:**
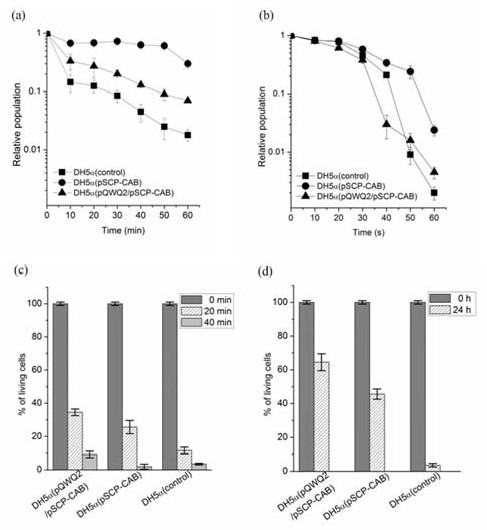
**Effect of heat shock (a), ultraviolet irradiation (b), acid (c) and osmotic pressure (d) on the survival of different *E. coli *strains**. Each experiment was performed at least three times. The error bars indicate standard deviations.

**Figure 5:**
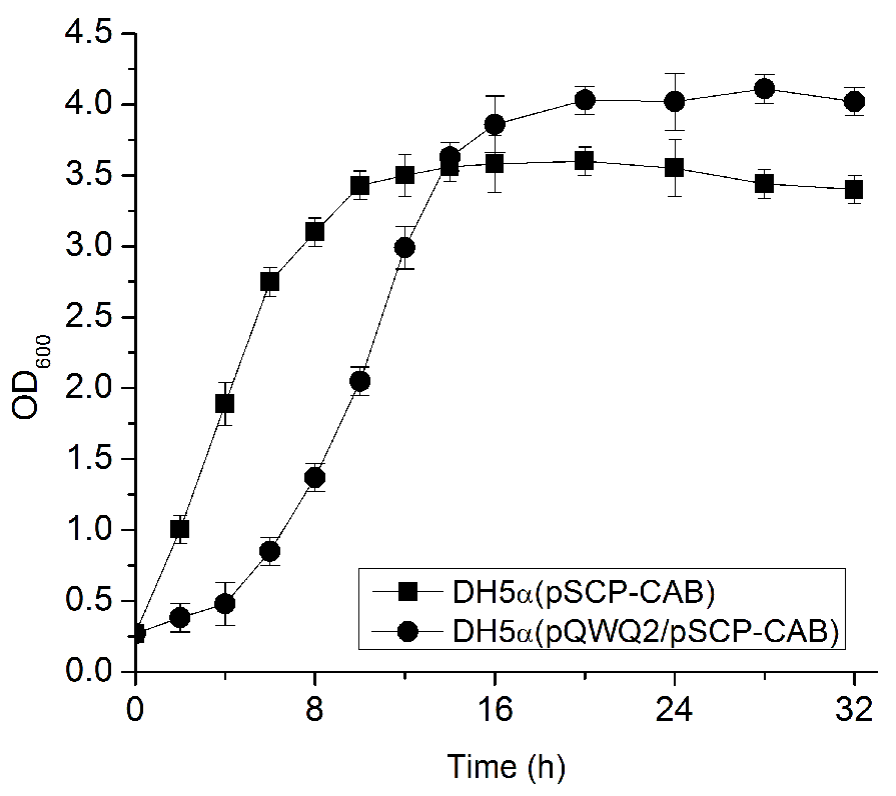
**Cell growth comparison of *E. coli *DH5α (pSCP-CAB) and *E. coli *DH5α (pQWQ2/pSCP-CAB)**. Growth was measured in LB medium with 20 g/L glucose without pH control.

Then, the engineered *E. coli *was used to investigate stress resistance capability. *E. coli *strain with PHB production or mobilization was confirmed to exhibit a changed stress capability (Fig. 4). When strains were incubated at 65°C in a water-bath, the PHB production strain, *E. coli *DH5α (pSCP-CAB), and PHB mobilization strain, *E. coli *DH5α (pQWQ2/pSCP-CAB), exhibited an increased heat resistance compared with the control strain (Fig. 4A). After 60 min of heating, about 30% and 7% of the initial number of cells remained alive, as compared to only 1.8% for the control strain. Likewise, *E. coli *DH5α (pSCP-CAB) and *E. coli *DH5α (pQWQ2/pSCP-CAB) showed an increased tolerance to UV irradiation compare to wild type *E. coli *(Fig. 4B). Acid resistance of the three strains was also studied: *E. coli *DH5α (pQWQ2/pSCP-CAB) exhibited a highest survival rate among the three strains (Fig. 4C). By 20 min of acid treatment, cell viability in *E. coli *DH5α (pQWQ2/pSCP-CAB), *E. coli *DH5α (pSCP-CAB) and *E. coli *DH5α control was reduced to 34.5%, 25.6% and 11.6% of the initial number of inoculated cells, respectively. By 40 min of acid treatment, cell viability was reduced to 9.4%, 3.2% and 1.8% of the initial number of inoculated cells, respectively. *E. coli *DH5α (pQWQ2/pSCP-CAB) also exhibited the highest survival rate when treated by a glucose-induced osmotic pressure (Fig. 4D). The viability of the *E. coli *DH5α control strain was especially affected by high concentration of glucose. Only about 2% of cells were alive when treated with 25% glucose. Interestingly, we found *E. coli *with PHB mobilization system exhibited a better growth compared with that with only PHB production system although *E. coli *DH5α (pQWQ2/pSCP-CAB) showed a long lag phase (Fig. 5).

## Discussion

Many studies showed that PHA biosynthesis would be promoted under nitrogen-deficient conditions in micro-organisms [23]. Under nitrogen deficient conditions, the metabolism of TCA cycle will be repressed, resulting in an increase of acetyl-CoA. Thus, the surplus of acetyl-CoA would be redirected to the PHA biosynthesis. While facing different stresses, such as low nutrient availability, especially carbon source limitation, these bacteria begin to mobilize PHA to conquer those unfavorable environments. In this study, we constructed a PHB mobilization system in *E. coli *using the stress induced promoter. The *rpoS *promoter in *E. coli *was confirmed previously to be induced under various stress conditions, such as cold shock, pH stress as well as cell density [20]. Some reports also pointed out that in *E. coli *the levels of sigma factor, RpoS, increase in response to starvation for carbon, nitrogen, or phosphate sources as well as for amino acids [21]. Here we confirmed that this stress induced promoter of *rpoS *can be induced under nitrogen limitation, which is a PHA accumulation condition in PHA producing micro-organisms. Thus, the PHB accumulation process in engineered *E. coli *under nitrogen limitation is similar as that in natural PHB producer.

In micro-organisms, PHA formation and mobilization is an important process for stress survival [24-27]. PHA formation provided the host with carbon and energy storage [2,28], while PHB mobilization is also of great importance. Previous studies indicated that incomplete PHB mobilization system, like a *phaZ *mutant (lacking PHB depolymerase) of *Azospirillum brasilense*, showed low stress endurance in various challenges [29,30]. In this study, we constructed a PHB mobilization system in engineered *E. coli*. We found that a stress induced PHB mobilization remarkably improved the carbon starvation tolerance of the *E. coli *host cell. Meanwhile, the PHB mobilization in engineered *E. coli *also provided the host with some other stress resistance. The mechanisms by which the PHA cycle favors stress alleviation are not yet fully understood [26,29]. We supposed that at least two reasons are responsible for the improved stress resistance in engineered *E. coli *strains. First, PHA mobilization may work at the small-molecule levels. It was found that a rise in ATP and guanosine tetraphosphate (ppGpp) levels was concomitant with PHA degradation in *Pseudomonas oleovorans *[30]. While the ppGpp was found to increase mRNA translation of the central stationary phase regulator *rpoS *[31], which up-regulates resistance to environmental stress [32-34]. Recently, it was found that the enhanced cross-tolerance to different stress agents during PHA-depolymerization in *P. oleovorans *is related to an increase in the intracellular concentration of RpoS [35]. Second, PHA mobilization may influence the chaperone protein levels. It was demonstrated that the large amount PHB accumulation in recombinant *E. coli *acted as a stress on the cells, which reduced the cells' ability to synthesize metabolic proteins and induced the expression of various protective proteins. Three heat shock proteins (GroEL, GroES, and DnaK) were significantly up-regulated in PHB-accumulating cells of *E. coli *as it was shown by proteome analysis [36]. The heat shock protein HspA was reported to be synthesized and bound to the PHB granule surface, indicating that *E. coli *also synthesizes protective proteins to reduce stress by binding these proteins to recombinant expressed inclusion bodies [37]. These protective proteins are helpful to the overall stress resistance of the host.

**Figure 6:**
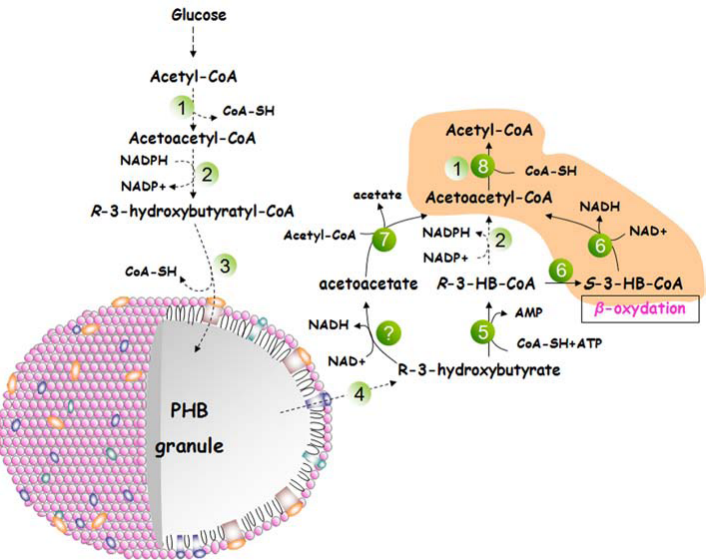
**Supposed metabolic network between PHB biosynthesis and mobilization in recombinant *E. coli *DH5α (pQWQ2/pSCP-CAB)**. Dashed lines represent enzymatic steps engineered in recombinant *E. coli*. Solid lines represent enzymatic steps existed in wild type *E. coli*. A question mark denotes unidentified enzyme in *E. coli*. ① *β*-ketothiolase, PhaA ② NADPH-dependent acetoacetyl-CoA reductase, PhaB ③ PHB synthase, PhaC ④ PHB depolymerase, PhaZ ⑤ acyl-CoA synthetase or thioesterase ⑥ 3-hydroxyacyl-CoA dehydrogenase, 3-hydroxybutyryl-CoA epimerase, FadB ⑦ acetoacetyl-CoA transferase, AtoA, AtoD ⑧ acetyl-CoA acetyltransferase, AtoB.

The secretion of 3HB in *E. coli *has also been observed in some previous experiments [9,38,39]. In this study, we observed the consumption of 3HB as carbon and energy source in engineered *E. coli*. The confirmation that 3HB can be converted into 3HB-CoA *in vivo *indicated the existence of a complete PHB cycle in engineered *E. coli*, from acetyl-CoA to PHB and from PHB to acetyl-CoA. This complete PHB mobilization is the reason of enhanced starvation and stress tolerance of the host. It was reported that a broad-substrate-ranged thioesterase YciA from *E. coli *mediated CoA-activation of the corresponding carboxylic acid, which supports the possibility of 3HB-CoA formation from 3HB in *E. coli *[15]. The PHB depolymerization rate is low in recombinant *E. coli*, thus we could not easily observe the 3HB accumulation under normal conditions. This is the first description that heterologous expression PHB mobilization system may change the stress resistance of the engineered *E. coli*. All previous experiments that concerned PHB mobilization and indicated altered stress resistance capability were done by knocking out key enzyme involved in PHB production in natural PHB production strains [29,30]. Taking advantage of the knowledge acquired previously and our findings in this study, we developed a schematic pathway to gain insight into the possible PHB mobilization routes in engineered *E. coli *(Fig. 6). The proposed pathway established in recombinant *E. coli in vivo *can mimic PHB cycle of the natural PHB producers: 3-HB, which proved to be actived to a CoA-link form, will be reversibly catalyzed by acetoacetyl-CoA reductase and *β*-ketothiolase to acetyl-CoA or will serve as a linker between the PHB mobilization with *β*-oxidation pathway.

## Conclusion

The PHB mobilization in *E. coli *that served as an intracellular energy and carbon storage changed the stress resistance of the host, which can enhance survival of *E. coli *when these sources are limited. Accumulation of PHB in succinate producing *E. coli *was confirmed to increase host resistance and showed a beneficial effect on succinate production (data submitted). Recombinant *E. coli *with PHB mobilization can also serve as immobilized cell factories for non-substrate/energy related biocatalysis and biotransformation since the cell number was not reduced even after long term incubation without carbon resource.

## Methods and materials

### Media and culture conditions

*Escherichia coli *strains were grown in Luria-Bertani medium (LB) at 37°C. Antibiotics were added to the corresponding cultures at a final concentration of 100 μg/mL ampicillin and 50 μg/mL spectinomycine for the maintenance of plasmids when necessary. For PHB production, *E. coli *pre-culture (0.5 mL) was inoculated into 50 mL LB or M9 medium supplemented with 2% (w/v) glucose as the sole carbon source in a 300-mL shake flask for 72 h. To analyze the PHB accumulation of recombinant *E. coli *under different C/N ratio (nitrogen limitation condition), various amount of nitrogen (0.2 g/L NH_4_Cl, 1 g/L NH_4_Cl, 2.5 g/L NH_4_Cl and 5 g/L NH_4_Cl) were used. The basal medium was 15.138 g/L Na_2_HPO_4_·H_2_O, 3 g/L KH_2_PO_4_, 0.5 g/L NaCl, 1 mg/L Thiamine VB_1_, 1 mM MgSO_4 _and 0.1 mM CaCl_2 _supplemented with 16 g/L glucose as carbon source.

### Construction of recombinant *E. coli *strains

DNA manipulation was performed by standard procedures [40]. Chromosomal DNA of *R. eutropha *was prepared by genomic DNA purification kit (Fermentas Inc.). PCR was carried out with an automatic thermal cycler (Takara Shuzo Co., Kyoto, Japan). Primer pair 5'- AAAGGATCCGCCTGCACAAAATTCCACCGTTGCTG and 5'-TTTGGGCCCCCCCTCGAGGTCGACGGTAT were used to amplify the stress-induced region (SIR) fragment from pQKZ100 [20]. The SIR fragment included *rpoS *promoter and the 5'-untranslated regulation region of *rpoS *gene. Primer pair 5'-TTTGGATCCCGACAGTAAGACGGGTAAGCCTGTTGATGAT and 5'-ACTGGGCCCGAGCTCCTTGAACGAATTGTTAGACATTATTTG were used to amplify the stable low copy number plasmid vector pCL1920 [41]. The two PCR products were digested with *Bam*HI and *Apa*I, and ligated to form plasmid pSCP. Fragment of 5.4 kb *Sma*I - *Eco*RI *phbCAB *operon from pBHR68 [42] was subcloned into pSCP, which resulted in plasmids pSCP-CAB. To construct the *phaZ*1 expression plasmid, the intact *phaZ*1 gene was amplified by PCR using the genomic DNA from *R. eutropha *as template with a pair of primers (5'-ATAAGCTTAAGGAGAATGCTCTACCAATTGCATGAGTT and 5'-ATCTCGAGTTACCTGGTGGCCGAGGCCT). The PCR product was digested with *Hin*dIII and *Xho*I and inserted into corresponding sites of pQKZ100, resulting plasmid pQWQ2.

### Stress endurance

After cultivation in M9 medium supplemented with 2% (w/v) glucose, cells were collected and washed twice by centrifugation at 4,000 *g *for 10 min and re-suspended in M9 medium without carbon source. The number of viable cells (CFU/mL) was determined by dilution plating prior to and at the end of each experiment (three replicates). For each experiment, the same initial number of cells was used (between 1 × 10^7 ^and 1 × 10^8 ^cells/mL). In heat resistance experiment, cells were incubated in a water-bath at 65°C for a total of 60 min. Samples (10 μL) were taken every 20 min to test the survival rate. The resistance test of UV irradiation was performed by placing 10 mL of cells in 90 mm Petri dishes and by exposing to short-wave UV irradiation (254 nm) from a Lourmat VL-6 LC UV lamp for 60 s. The UV treated cells were collected and re-suspended in potassium phosphate buffer (0.06 M, pH 6.8) for checking the surviving rate. In the acid resistance experiment, cells were maintained in 0.05 M potassium phosphate buffer (adjusted pH value to 3.0). The sensitivity of cells to osmotic pressure was estimated by adding 25 mL 50% glucose (w/v) solution to 25 mL cell suspension to give a final glucose concentration of 25% (w/v).

### Starvation experiment

After cultivation in M9 medium supplemented with 2% (w/v) glucose, cells were collected and washed twice by centrifugation at 4,000 *g *for 10 min and re-suspended in M9 medium without carbon source (pH 6.8). Cells were then incubated on a shaker at 200 r.p.m for 30 days under starvation conditions [43]. Bacterial density (CFU/mL) was determined by dilution plating (three replicates). Cells were diluted to about 1 × 10^7 ^cells/mL.

### Determination of PHB and 3HB

Samples (5 mL) were taken out from culture medium every 2 or 4 hours (before 72 h) or 4 days (after 72 h), and were lyophilized overnight. The PHB content was determined by gas chromatography (GC) after methanolysis of the lyophilized cells in the presence of 15% sulfuric acid. Purified PHB polymer was obtained by chloroform extraction for 72 h and ethanol precipitation at room temperature [44]. Culture supernatants were used to detect the 3HB monomer by high-pressure liquid chromatography (HPLC) on a reversed-phase column (Waters C18; 5 mm, 4.6 mm by 15 cm). 0.02 M NaH_2_PO_4 _(pH 2~3) solution was used as the mobile phase at the flow rate of 0.8 mL/min.

### 3-HA-CoA synthase activity

The acyl-CoA synthetase activity was measured in 100 mM potassium phosphate buffer (pH 7.5) containing 5 mM ATP, 10 mM organic acid, 1.25 mM CoASH, 5 mM dithiothreitol (DTT), 5 mM magnesium chloride. To start the reaction, enzyme sample (20 μL) was added to the assay mixture to form a final volume of 200 μL. After 15 min of incubation at 30°C, the reaction was quenched by adding 20 μL of 10% (v/v) formic acid. 3HB consumption was detected by HPLC as described above.

## Competing interests

The authors declare that they have no competing interests.

## Authors' contributions

QW carried out most of the experiments and wrote the manuscript. QW and HY carried out the resistance experiment. QW, YX, and ZK constructed the plasmids and strains. QQ conceived of the study, participated in its design, and drafted the manuscript. All authors read and approved the final manuscript.

## Supplementary Material

Additional file 1 Confirmation of PHB accumulation in engineered *E. coli *DH5α (pSCP-CAB) and cell survival rate and PHB mobilization of recombinant *E. coli *DH5α for long term starvation in potassium phosphate buffer. Click here for file

## References

[B1] Anderson AJ, Dawes EA (1990). Occurrence, metabolism, metabolic role, and industrial uses of bacterial polyhydroxyalkanoates. Microbiol Rev.

[B2] Dawes EA, Senior PJ (1973). The role and regulation of energy reserve polymers in micro-organisms. Adv Microb Physiol.

[B3] Bergersen FJ, Peoples MB, Turner GL (1991). A Role for poly-β-hydroxybutyrate in Bacteroids of Soybean Root Nodules. Proceedings.

[B4] Kadouri D, Jurkevitch E, Okon Y, Castro-Sowinski S (2005). Ecological and agricultural significance of bacterial polyhydroxyalkanoates. Crit Rev Microbiol.

[B5] Taidi B, Mansfield DA, Anderson AJ (1995). Turnover of poly (3-hydroxybutyrate)(PHB) and its influence on the molecular mass of the polymer accumulated by *Alcaligenes eutrophus *during batch culture. FEMS Microbiol Lett.

[B6] Slater SC, Voige WH, Dennis DE (1988). Cloning and expression in *Escherichia coli *of the *Alcaligenes eutrophus *H16 poly-beta-hydroxybutyrate biosynthetic pathway. J Bacteriol.

[B7] Senior PJ, Dawes EA (1973). The regulation of poly-beta-hydroxybutyrate metabolism in *Azotobacter beijerinckii*. Biochem J.

[B8] Oeding V, Schlegel HG (1973). Beta-ketothiolase from *Hydrogenomonas eutropha *H16 and its significance in the regulation of poly-beta-hydroxybutyrate metabolism. Biochem J.

[B9] Uchino K, Saito T, Jendrossek D (2008). Poly(3-hydroxybutyrate) (PHB) depolymerase PhaZa1 is involved in mobilization of accumulated PHB in *Ralstonia eutropha *H16. Appl Environ Microbiol.

[B10] Saegusa H, Shiraki M, Kanai C, Saito T (2001). Cloning of an intracellular Poly[D(-)-3-Hydroxybutyrate] depolymerase gene from *Ralstonia eutropha *H16 and characterization of the gene product. J Bacteriol.

[B11] Jendrossek D, Schirmer A, Schlegel HG (1996). Biodegradation of polyhydroxyalkanoic acids. Appl Microbiol Biotechnol.

[B12] Aneja P, Zachertowska A, Charles TC (2005). Comparison of the symbiotic and competition phenotypes of *Sinorhizobium meliloti *PHB synthesis and degradation pathway mutants. Can J Microbiol.

[B13] Ruth K, de Roo G, Egli T, Ren Q (2008). Identification of two acyl-CoA synthetases from *Pseudomonas putida *GPo1: one is located at the surface of polyhydroxyalkanoates granules. Biomacromolecules.

[B14] Sandoval A, Arias-Barrau E, Bermejo F, Canedo L, Naharro G, Olivera ER, Luengo JM (2005). Production of 3-hydroxy-n-phenylalkanoic acids by a genetically engineered strain of *Pseudomonas putida*. Appl Microbiol Biotechnol.

[B15] Zhuang Z, Song F, Zhao H, Li L, Cao J, Eisenstein E, Herzberg O, Dunaway-Mariano D (2008). Divergence of function in the hot dog fold enzyme superfamily: the bacterial thioesterase YciA. Biochemistry.

[B16] Cai GQ, Driscoll BT, Charles TC (2000). Requirement for the enzymes acetoacetyl coenzyme A synthetase and poly-3-hydroxybutyrate (PHB) synthase for growth of *Sinorhizobium meliloti *on PHB cycle intermediates. J Bacteriol.

[B17] Lee SY, Lee KM, Chan HN, Steinbuchel A (1994). Comparison of recombinant *Escherichia coli *strains for synthesis and accumulation of poly-(3-hydroxybutyric acid) and morphological changes. Biotechnol Bioeng.

[B18] Li R, Chen Q, Wang PG, Qi Q (2007). A novel-designed *Escherichia coli *for the production of various polyhydroxyalkanoates from inexpensive substrate mixture. Appl Microbiol Biotechnol.

[B19] Shiraki M, Endo T, Saito T (2006). Fermentative production of (R)-(-)-3-hydroxybutyrate using 3-hydroxybutyrate dehydrogenase null mutant of *Ralstonia eutropha *and recombinant Escherichia coli. J Biosci Bioeng.

[B20] Kang Z, Wang Q, Zhang H, Qi Q (2008). Construction of a stress-induced system in *Escherichia coli *for efficient polyhydroxyalkanoates production. Appl Microbiol Biotechnol.

[B21] Hengge-Aronis R (2002). Signal transduction and regulatory mechanisms involved in control of the sigma(S) (RpoS) subunit of RNA polymerase. Microbiol Mol Biol Rev.

[B22] Madison LL, Huisman GW (1999). Metabolic engineering of poly(3-hydroxyalkanoates): from DNA to plastic. Microbiol Mol Biol Rev.

[B23] Durner R, Witholt B, Egli T (2000). Accumulation of Poly[(R)-3-hydroxyalkanoates] in *Pseudomonas oleovorans *during growth with octanoate in continuous culture at different dilution rates. Appl Environ Microbiol.

[B24] James BW, Mauchline WS, Dennis PJ, Keevil CW, Wait R (1999). Poly-3-hydroxybutyrate in *Legionella pneumophila*, an energy source for survival in low-nutrient environments. Appl Environ Microbiol.

[B25] Pettinari MJ, Vazquez GJ, Silberschmidt D, Rehm B, Steinbuchel A, Mendez BS (2001). Poly(3-hydroxybutyrate) synthesis genes in *Azotobacter *sp. strain FA8. Appl Environ Microbiol.

[B26] Kadouri D, Jurkevitch E, Okon Y (2003). Involvement of the reserve material poly-beta-hydroxybutyrate in *Azospirillum brasilense *stress endurance and root colonization. Appl Environ Microbiol.

[B27] Alvarez B, Lopez MM, Biosca EG (2008). Survival strategies and pathogenicity of *Ralstonia solanacearum *phylotype II subjected to prolonged starvation in environmental water microcosms. Microbiology.

[B28] Lee SY (1996). Bacterial polyhydroxyalkanoates. Biotechnol Bioeng.

[B29] Kadouri D, Jurkevitch E, Okon Y (2003). Poly beta-hydroxybutyrate depolymerase (PhaZ) in *Azospirillum brasilense *and characterization of a phaZ mutant. Arch Microbiol.

[B30] Ruiz JA, Lopez NI, Fernandez RO, Mendez BS (2001). Polyhydroxyalkanoate degradation is associated with nucleotide accumulation and enhances stress resistance and survival of *Pseudomonas oleovorans *in natural water microcosms. Appl Environ Microbiol.

[B31] Brown L, Gentry D, Elliott T, Cashel M (2002). DksA affects ppGpp induction of RpoS at a translational level. J Bacteriol.

[B32] Lange R, Hengge-Aronis R (1991). Identification of a central regulator of stationary-phase gene expression in *Escherichia coli*. Mol Microbiol.

[B33] Sarniguet A, Kraus J, Henkels MD, Muehlchen AM, Loper JE (1995). The sigma factor sigma s affects antibiotic production and biological control activity of *Pseudomonas fluorescens *Pf-5. Proc Natl Acad Sci.

[B34] Ramos-Gonzalez MI, Molin S (1998). Cloning, sequencing, and phenotypic characterization of the rpoS gene from *Pseudomonas putida *KT2440. J Bacteriol.

[B35] Ruiz JA, Lopez NI, Mendez BS (2004). rpoS gene expression in carbon-starved cultures of the Polyhydroxyalkanoate-accumulating species *Pseudomonas oleovorans*. Curr Microbiol.

[B36] Han MJ, Yoon SS, Lee SY (2001). Proteome analysis of metabolically engineered *Escherichia coli *producing Poly(3-hydroxybutyrate). J Bacteriol.

[B37] Tessmer N, Konig S, Malkus U, Reichelt R, Potter M, Steinbuchel A (2007). Heat-shock protein HspA mimics the function of phasins sensu stricto in recombinant strains of *Escherichia coli *accumulating polythioesters or polyhydroxyalkanoates. Microbiology.

[B38] Lee SY, Lee Y, Wang F (1999). Chiral compounds from bacterial polyesters: sugars to plastics to fine chemicals. Biotechnol Bioeng.

[B39] Gao HJ, Wu Q, Chen GQ (2002). Enhanced production of D-(-)-3-hydroxybutyric acid by recombinant *Escherichia coli*. FEMS Microbiol Lett.

[B40] Sambrook J, Russell DW (2001). Molecular Cloning: A Laboratory Manual.

[B41] Lerner CG, Inouye M (1990). Low copy number plasmids for regulated low-level expression of cloned genes in *Escherichia coli *with blue/white insert screening capability. Nucleic Acids Res.

[B42] Spiekermann P, Rehm BH, Kalscheuer R, Baumeister D, Steinbuchel A (1999). A sensitive, viable-colony staining method using Nile red for direct screening of bacteria that accumulate polyhydroxyalkanoic acids and other lipid storage compounds. Arch Microbiol.

[B43] Tal S, Okon Y (1985). Production of the reserve material poly- beta-hydroxybutyrate and its function in *Azospirillum brasilense *Cd. Can J Microbiol.

[B44] Qi Q, Rehm BH (2001). Polyhydroxybutyrate biosynthesis in *Caulobacter crescentus*: molecular characterization of the polyhydroxybutyrate synthase. Microbiology.

