# Supplementary material for: Complete PHB mobilization in Escherichia coli enhances the stress tolerance: a potential biotechnological application
Source: Microb Cell Fact. 2009 Aug 31;8:47. doi: 10.1186/1475-2859-8-47 (PMC2746179; doi:10.1186/1475-2859-8-47)
Supplement: Additional file 1 — Confirmation of PHB accumulation in engineered E. coli DH5α (pSCP-CAB) and cell survival rate and PHB mobilization of recombinant E. coli DH5α for long term starvation in potassium phosphate buffer. [file 1475-2859-8-47-S1.doc]

**Supplementary Material:**

**Complete PHB mobilization in *Escherichia coli* enhances the stress tolerance: a potential biotechnological application**

Qian Wang, Hongmin Yu, Yongzhen Xia, Zhen Kang, Qingsheng Qi*

**State Key Laboratory of Microbial Technology, National Glycoengineering Research Center,** School of Life Science

Shandong University, Jinan, 250100 P. R. China

* Corresponding author: Tel & Fax: +86-531-88365628

**Confirmation of PHB accumulation in engineered *E. coli* DH5α (pSCP-CAB)**


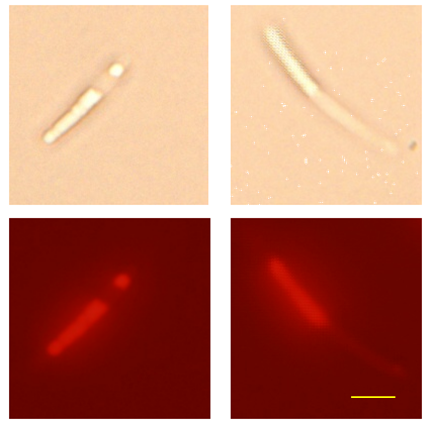


**Figure S1: Fluorescence microscopy of *E.coli* DH5α (pSCP-CAB).** Cells were stained with Nile Red after cultivated in M9 medium with glucose for 40h. bar 1 μm

**Cell survival rate and PHB mobilization of recombinant *E. coli* DH5α for long term starvation in potassium phosphate buffer**

Cell number of all strains in potassium phosphate buffer rapidly decreased from the onset of the starvation except *E. coli* DH5α (pQWQ2/pSCP-CAB). At the end of the starvation experiment (32 days), almost all the cells without a PHB mobilization system was died while the cells with a PHB mobilization system can still survive (Figure S2A). Meanwhile, the PHB content in PHB mobilizing cells decreased 20%, indicating the necessary of nitrogen for the survival and multiplication during the starvation (Figure S2B).

**Figure S2: Effect of PHB mobilization on the survival of starved *E. coli* strains in potassium phosphate buffer.**
